# Patterns of cancer cell sphere formation in primary cultures of human oral tongue squamous cell carcinoma and neck nodes

**DOI:** 10.1186/s12935-014-0143-3

**Published:** 2014-12-21

**Authors:** Saira Saleem, Arif Jamshed, Saima Faisal, Raza Hussain, Muhammad Tahseen, Asif Loya, Chris Sutton

**Affiliations:** Basic Sciences Research Department, Shaukat Khanum Memorial Cancer Hospital and Research Center, 7-A Block R-3, Johar Town, Lahore, 54000 Pakistan; Department of Radiation Oncology, Shaukat Khanum Memorial Cancer Hospital and Research Center, 7-A Block R-3, Johar Town, Lahore, 54000 Pakistan; Clinical Research Office, Shaukat Khanum Memorial Cancer Hospital and Research Center, 7-A Block R-3, Johar Town, Lahore, 54000 Pakistan; Department of Surgical Oncology, Shaukat Khanum Memorial Cancer Hospital and Research Center, 7-A Block R-3, Johar Town, Lahore, 54000 Pakistan; Department of Pathology, Shaukat Khanum Memorial Cancer Hospital and Research Center, 7-A Block R-3, Johar Town, Lahore, 54000 Pakistan; Institute of Cancer Therapeutics, University of Bradford, Tumbling hill street, Bradford, BD7 1BD United Kingdom

**Keywords:** Oral tongue squamous cell carcinoma, Lymph node metastasis, Primary culture, Cancer cell sphere, Cancer stem cells, *in vitro* assay

## Abstract

Recently a sub-population of cells with stem cell characteristics, reported to be associated with initiation, growth, spread and recurrence, has been identified in several solid tumors including oral tongue squamous cell carcinoma (OTSCC). The aim of our pilot study was to isolate CD44+ cancer stem cells from primary cultures of OTSCC and neck node Level I (node-I) biopsies, grow cell spheres and observe their characteristics in primary cultures. Parallel cultures of hyperplastic lesions of tongue (non-cancer) were set up as a control. Immunohistochemistry was used to detect CD44/CD24 expression and magnetic activated cell sorting to isolate CD44+ cell populations followed by primary cell culturing. Both OTSCC and node-I biopsies produced floating spheres in suspension, however those grown in hyperplastic and node-I primary cultures did not exhibit self-renewal properties. Lymph node metastatic OTSCC, express higher CD44/CD24 levels, produce cancer cell spheres in larger number and rapidly (24 hours) compared to node negative OTSCC (1 week) and non-cancer specimens (3 weeks). In addition, metastatic OTSCC have the capacity for proliferation for up to three generations in primary culture. This *in vitro* system will be used to study cancer stem cell behavior, therapeutic drug screening and optimization of radiation dose for elimination of resistant cancer cells.

**Electronic supplementary material:**

The online version of this article (doi:10.1186/s12935-014-0143-3) contains supplementary material, which is available to authorized users.

## Introduction

The incidence of head and neck squamous cell carcinoma (HNSCC) varies worldwide but is more prevalent in South Asia [1,2]. In Pakistan, the factors contributing to increased incidence in oral cancer were life style habits of smoking, betel quid chewing, and other addictive substances [3]. For all age groups and both genders, lip and oral cavity malignancies were the third most prevalent (5.88% of the 63,881 cases registered from 1994–2013 - www.shaukatkhanum.org.pk). The commonly observed presence of neck lymph node metastasis (LNM) in oral squamous cell carcinoma (OSCC) patients signifies the regional spread of the disease. Despite various treatment options for OSCC, the survival rates are poor, which is in part attributed to the limited understanding of the resistant cancer cells which are now termed cancer stem cells (CSCs) or tumor initiating cells (TICs).

The hypothesis that only a sub-fraction of cells within a tumor, sharing stem-like characteristics, can regenerate the heterogeneous tumor, promote metastasis and recurrence, is gaining importance and is termed cancer stem cell theory [4]. The first evidence for the existence of CSCs was reported in blood borne cancer based on expression of CD34 + CD38- markers [5]. This was followed by their identification in solid tumor cancers including breast (CD44 + CD24-) [6,7], brain (CD133+) [8], prostate (CD44+) [9] and pancreatic tumor (CD44 + CD24 + ESA+) [10].

Identification of CD44+ head and neck cancer stem cells (HNCSC) is a relatively recent discovery following isolation from a mouse xenograft model of HNSCC [11]. Clonogenic assays produce a gradient of change in morphology and compactness of cells constituting different colonies, namely holoclones, meroclones and paraclones consisting of tightly, loosely and sparsely packed cells, respectively which can be used to evaluate CSC properties [12]. Immortalised cell lines, however, will have acquired cellular and phenotypic changes and may not accurately represent molecular and cellular events occurring in *in vivo* tumors. In the present study, we used primary cell sources isolated from human tongue tissue biopsies to study CSC properties.

## Materials and methods

All chemicals obtained from Sigma unless specified otherwise.

### Patient selection

Ethical approval of the study was obtained from the Internal Review Board on Human Research at Shaukat Khanum Memorial Cancer Hospital and Research Center (SKMCH&RC), Pakistan. A total of 8 clinical tissue specimens were obtained after getting consent from patients undergoing surgery during the year 2013. Partial glossectomy was performed to collect five primary tongue tumors and three hyperplastic growths on tongue as non-cancer controls. Where there was unilateral neck dissection or bilateral modified radical neck dissection undertaken alongside glossectomy, five node-I specimens were collected. Freshly resected specimens were acquired by a pathologist and 3mm^3^ of biopsy collected in RMPI medium with 5× antibiotic for cell culture and isolation of cells. Remaining tissue was immediately preserved either by fixing in formalin for embedding into paraffin wax (for histopathological diagnosis) or stored at −80°C in 1×PBS (phosphate-buffered saline). The presence of hyperplastic or neoplastic cells in the acquired specimen was confirmed by two clinical pathologists. The clinical and pathological characteristics of patients are summarized in Table 1.

**Table 1:** **Characteristics of study subjects with tongue cancer or hyperplasia**

**Patient**	**Gender**	**Age**	**Tobacco use**	**Alcohol use**	**Betel nut use**	**Primary tumor site**	**Histologic grading**	**T classification**	**Lymph node involvement**	**Distant metastasis**	**Prior radiation**	**Prior chemo**
1	F	60	No	No	No	Tongue	Hyperplasia	pT0	pN0	M0	No	No
2	M	60	No	No	No	Tongue	ND	pTx	pN1a	M0	No	No
3	F	55	No	No	1-2 times a day. Started at the age of 18. Quit since past 5 months.	Tongue	G2	pT2	pN0	M0	No	No
4	M	49	ocassional smoking. Started at the age of 20. Quit for the past 8 months.	No	3-4 times a day. Started at the age of 16. Quit since past 8 months.	Tongue	G1	pT1	pN0	M0	Yes	Cisplatin + Gemcitabine 2x
5	F	26	No	No	1 sachet/day. Started at the age of 6. Quit since past one year.	Tongue	G2	pT2	pN0	M0	No	No
6	M	46	No	No	No	Tongue	Hyperplasia	pT0	Not resected	M0	No	No
7	M	25	No	No	No	Tongue	G2	pT1	pN0	M0	No	Cisplatin + Gemcitabine 2x
8	F	56	2-3 cigrettes/day. Started at the age of 7. Still somkes.	No	No	Tongue	Hyperplasia	pT0	Not resected	M0	No	No

p: pathologic.

T0: No evidence of tumor.

T1: Tumor 2cm or less in greatest dimension.

T2: Tumor more than 2cm but not more than 4cm in greatest dimension.

ND: Not detected.

N0: No regional lymph node metastasis.

N1a: Metastasis in a single ipsilateral lymph node, 3 cm or less in greatest dimension.

M0: No evidence of distant metastasis.

G1: Well differentiated.

G2: Moderately differentiated.

### Immunohistochemistry

Sections (4 μm) were de-paraffinized in xylene, rehydrated for CD44 and CD24 detection. The sections were treated with hydrogen peroxide (H_2_O_2_; 3% 10 min) at room temperature (RT), washed in distilled water (DW), treated with microwave heating for 15 min in a citrate buffer (pH 9.0) for antigen retrieval, washed in DW and treated with PBS for 5 min. Non-specific binding was blocked by incubation with blocking reagent (Dako) for 5 min at RT, in a wet chamber. Incubation with a monoclonal antibody against all human CD44 isoforms (1:50 dilution, R&D Systems) or all CD24 isoforms (1:1000 dilution, R&D Systems) was carried out for 35 min at 37°C. The immunoreactivity was detected using the Dako Envision kit with peroxidase activity using 3, 3’-diaminobenzidine (DAB) as substrate. Finally, the sections were counterstained with a hematoxylin, dehydrated, cleared in xylene and mounted with Eukitt (DeltaLab). Immunohistochemistry negative controls were prepared by omitting the primary antibody.

### Tumor sphere culturing

Each biopsy was proteolytically digested to generate single-cell suspensions. Briefly, specimens were cut into small fragments, minced with a sterile scalpel, and immersed in Dulbecco’s Modified eagles Medium (DMEM) containing 40 Units Dispase II /mL. The combination was incubated at 37°C for up to 3 hours and with mixing every 15 minutes to enable complete digestion. The resulting single-cell suspension was filtered through 40 μm nylon mesh and washed twice with HBSS/2% HICS (Hank’s buffered salt solution in heat inactivated calf serum). Primary cultures for each type of fresh specimen (OTSCC tumor, hyperplastic and neck node) were set up separately by the method of Harper *et al.* without the use of feeder cells [13]. Briefly, specimens were collected in RM+ [consisting of a 3:1 ratio of DMEM:F12 with 10% fetal bovine serum (FBS), 1% glutamine, 0.4 μg hydrocortisone, transferrin 5 μg⁄mL, insulin 5 μg⁄mL, EGF 10 ng⁄mL, 1× antibiotic⁄antimycotic mixture] and incubated overnight at 4°C. The samples were washed (3×) in fresh RM+ medium with vortexing to remove any debris. Approximately 1mm^2^ pieces were placed in culture flask coated with thin layer of autoclaved 0.1% agarose or agar. This was followed by addition of RM+ medium and culture flasks were placed in a humid incubator at 37°C and 5% CO_2_ with the medium changed once per week. When outgrowing cells approached confluence and floating spheres were formed, they were collected by centrifugation.

### Magnetic bead affinity cell sorting (MACS) of CD44+ and CD44- cells

Collected spheres from OTSCC tumor, hyperplastic and neck node cultures were dissociated with 40 Units Dispase II /mL of DMEM incubation and vortexed for 15 minutes to form single-cell suspension. Following, filtering and washing, a MagCellect™ cell isolation kit (R&D Systems) was used to isolate CD44+ and CD44- cells from each as per the manufacturer’s instructions.

### CD44+ and CD44- cell culture

Isolated CD44+ and CD44- cells were cultured without feeder cells (fibroblasts or amniotic membrane) in RM+ medium to test their ability for forming spheres potentially harboring CSCs. The cells were passed through a 40 μm filter, and 10 mL of medium containing 2 × 10^4^ cells was added to 25 mm^2^ culture flasks coated with 0.1% agarose or agar. After 1–2 weeks culture flasks were visually assayed for the formation of floating spheres.

### Stem cellness self-renewal assay

The spheres were collected by centrifugation and digested to single-cell suspensions using Dispase II (20U/mL of DMEM). After passing through a 40 μm filter, 250 cells/cm^2^ cells in 10 mL of medium was added to non-adherent (0.1% agarose coated) or adherent culture flasks for daughter sphere formation and colony formation, respectively. The floating spheres and colonies were observed using phase contrast microscope (Labomed TCM 400). The viability of the cells was also confirmed by Trypan blue exclusion method.

## Results

### CD44/CD24 expression and sphere formation in primary cultures of tongue and node biopsies

**Figure 1:**
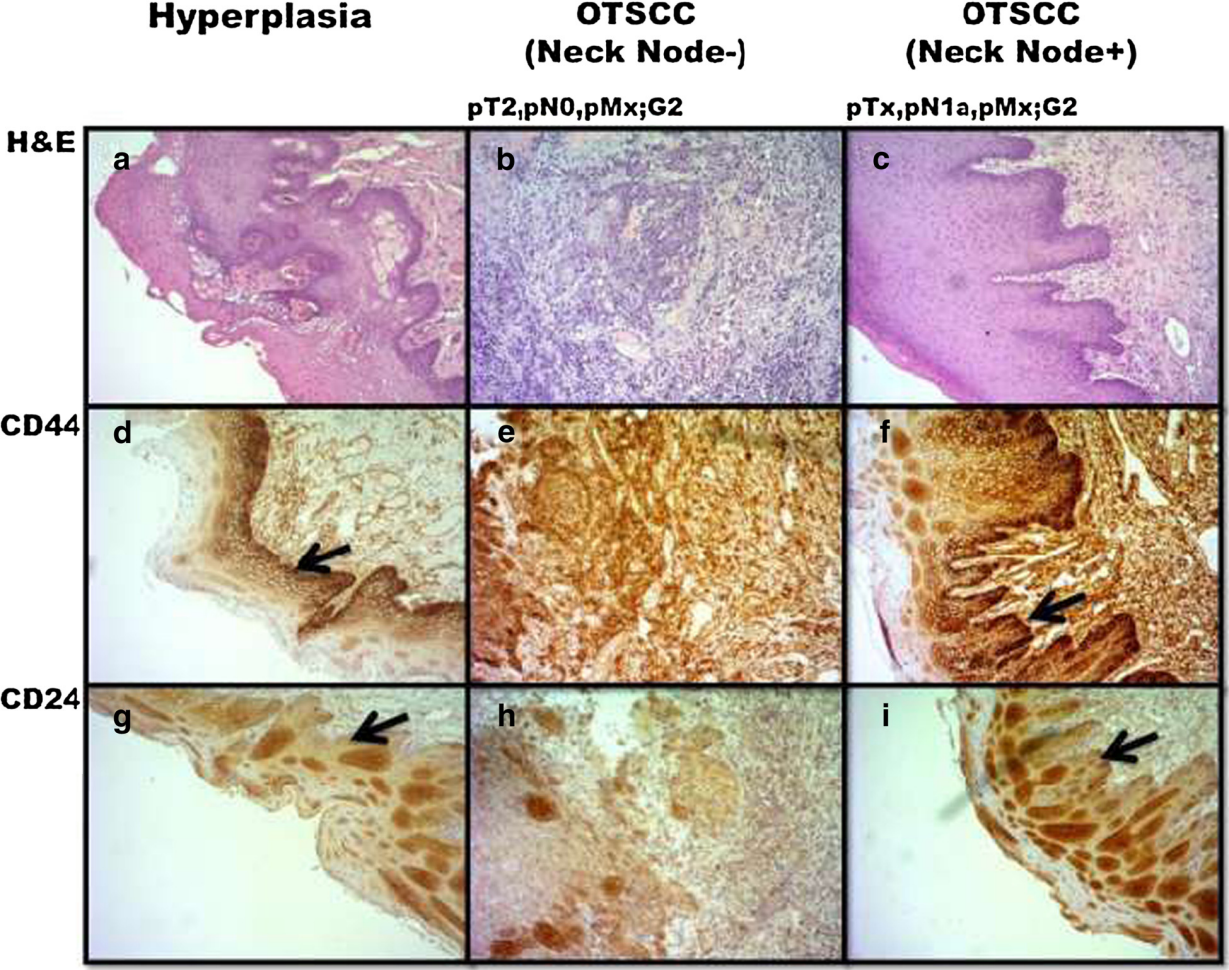
**Tumor grading and expression of CD44 and CD24 in selected tongue tissues.** Top panel **(a-c)**: H&E staining for diagnosis and grading of tongue lesion or tumors. Middle panel **(d-f)**: CD44 staining. Lower panel **(g-i)**: CD24 staining. The cells in the basal layer expressing CD44 (black arrows) are negative for CD24 (black arrows). Magnification 40x.

**Figure 2:**
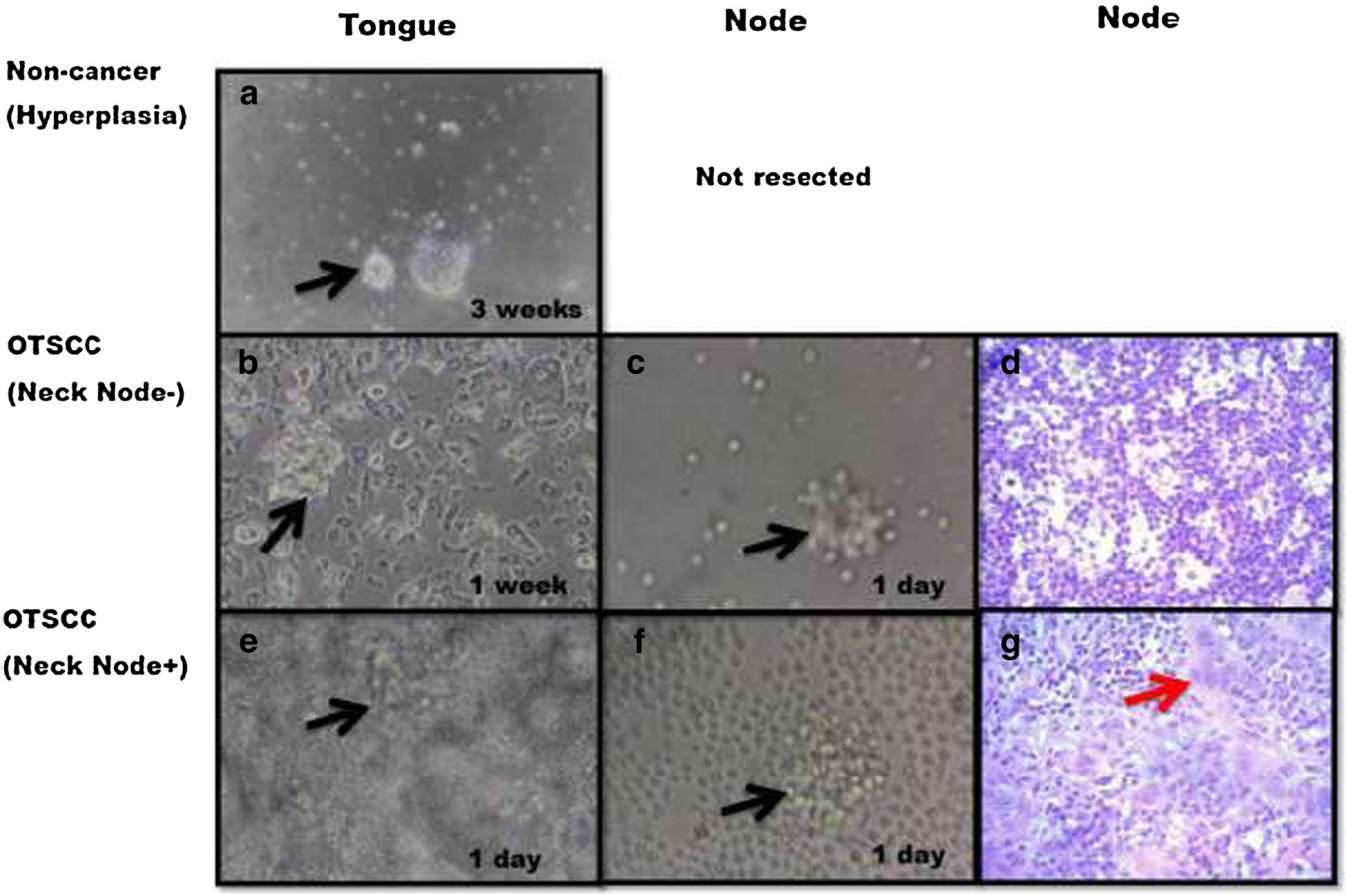
**Representative phase contrast images of spheres (black arrows) formed in suspension in primary cultures of OTSCC (a,b,e) and neck nodes (c,f) in growth factor supplemented medium in non-adhesive 0.1% agarose coated plastic ware and H&E staining of neck nodes (d,g).** **(a)** created and survived for 3 weeks. Daughter spheres were not formed. **(b)** created within 1 week, survived for 3 weeks. Re-passaged for 2 generations. **(c)** spheres formed in primary cultures of negative neck nodes within 24hours. Daughter spheres were not formed. **(d)** H&E staining of negative neck node **(e)** spheres created within 24 hours and survived for several weeks. Re-passaged for 3 generations. **(f)** spheres formed in primary cultures of positive neck nodes within 24hours. Daughter spheres were not formed. **(g)** H&E staining of positive neck node. Red arrow pointing to malignant cells. Magnification 40x.

Immunohistochemistry of tissue sections indicated increased CD44 expression (Figure 1a-c) through hyperplastic to neck node negative and neck node positive OTSCC. The cells in the basal layer expressing CD44 (Figure 1d-f) were negative for CD24 (Figure 1g-i) suggesting differential expression. The biopsies from hyperplastic, non-metastatic (neck node negative) and metastatic (neck node positive) OTSCC were incubated in conditioned media to observe their sphere formation patterns in primary cell culture (Table 2). The proliferation of cells was slowest in hyperplastic cells (Figure 2a), faster in non-metastatic OTSCC (Figure 2b) and fastest in lymph node metastatic OTSCC (Figure 2e), with pathologically negative and positive node-I (Figure 2d and g) tissues generating floating spheres within 24 hours (Figure 2c and f).

**Table 2:** **Number and size of spheres or spheroids in primary cultures and MACS isolated CD44+ cell cultures**

	**Primary cell spheres**					**CD44+ cell spheres**				
**Tissue type**		**Parent spheres**	**Passage 1**	**Passage 2**	**Passage 3**	**Parent spheres**	**Passage 1**	**Passage 2**	**Passage 3**	**Passage 4**
**H**	**Duration Number Cells/sphere**	3 weeks	None	None	None	Cluster of cells 10-15	None	None	None	None
		15-20								
		10-30								
**OTSCC (Neck Node -)**	**Duration Number Cells/sphere**	1 week	1 week	1 week	None	1 week	1 week	1 week	1 week	None
		30-40	15-20	10-15		20-30	20-30	10-15	Up to 10	
		20-50	10-25	up to 10		15-25	10-15	up to 10	3-5	
**OTSCC (Neck Node+)**	**Duration Number Cells/sphere**	1 day	1 day	1 day	1 day	1 day	1 day	1 day	1 day	None
		40-50	30-40	15-20	10-15	30-40	30-40	15-25	Up to 10	
		50-100	50-100	20-50	Up to 10	20-30	15-25	10-15	3-5	

Cells plated at a density of 250/cm^2^ in 10mL of conditioned media in 0.1% agarose or agar coated non-adhesive culture flasks. Triplicate analyses were done from each sample type.

### CD44+ cells primary culture

**Figure 3:**
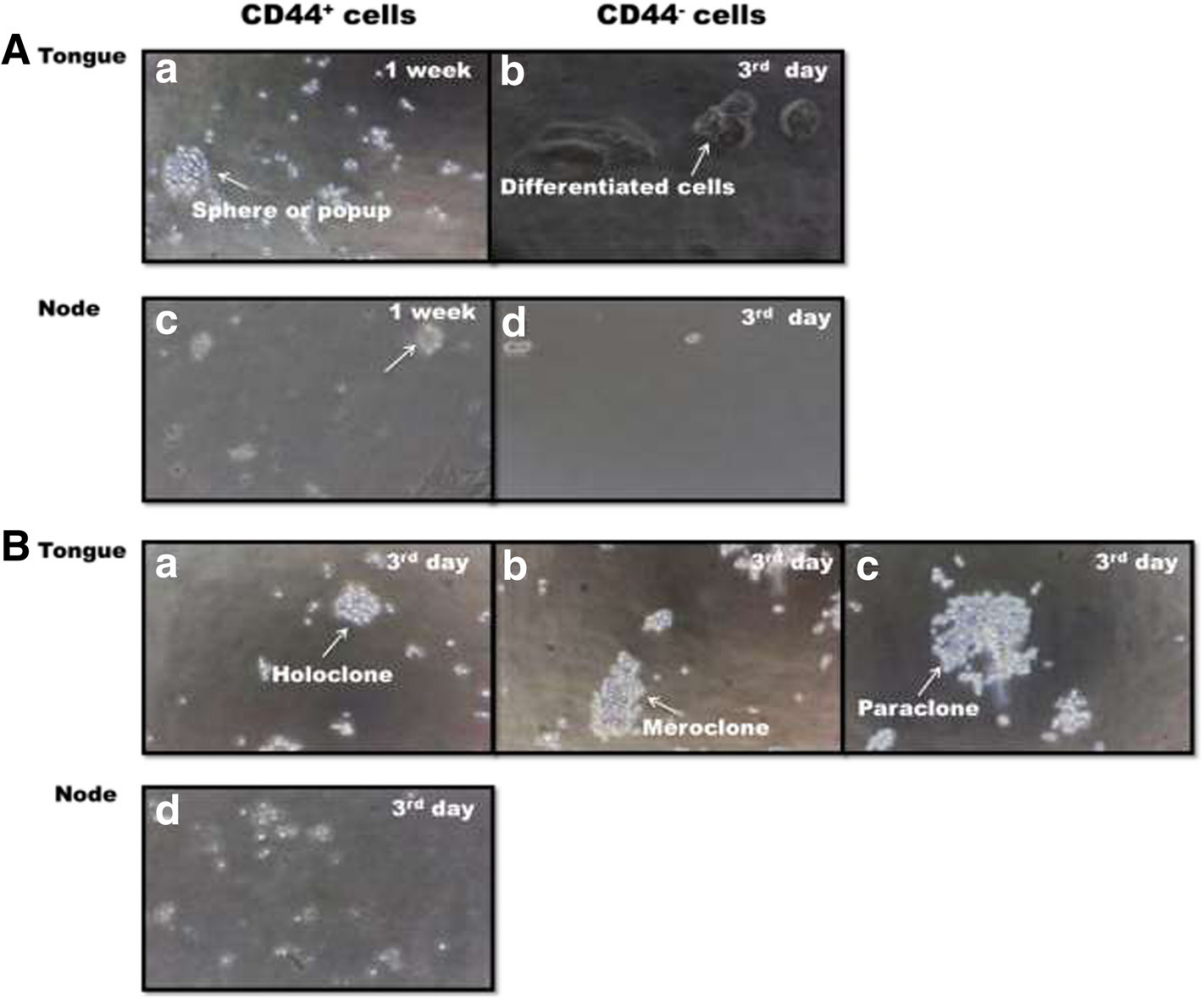
**Sphere formation by CD44+ cells isolated from tongue and neck node tissues. A:** CD44+ cells from tumor and neck node tissue (Magnetic activated bead isolation followed by cell culture in conditioned media in non-adhesive 0.1% agarose coated plastic ware. (a) The proliferating CD44+ cells from OTSCC (lymph node metastatic and non-metastatic) formed spheres or spheroids (b) CD44- cells differentiated during 1 week (c) CD44+ cells isolated from neck nodes (Level I) (d) CD44- cells from neck nodes (Level I) **B:** Clonogenic assay for self-renewal. Purified CD44+ cells were plated at 250cells/mm^2^ and cultured for 1 week. (a) holoclone comprising of compact cells (b) meroclone containing loosely arranged cells on adhesive plastic ware (c) paraclone comprising of sparsely arranged cells on adhesive plastic ware (d) No colonies were formed in nodes secondary cultures. No cell spheres were formed from non-cancer tissues (not shown). Magnification 40x.

Purified CD44+ cells exhibited much higher sphere-forming abilities than the CD44- counterparts. The floating cancer cell spheres were generated within 1 week from CD44+ cells (Figure 3A,a), whereas CD44- cells started to differentiate by 3rd day of the culture (Figure 3A, b). The cells isolated from hyperplastic or non-cancer tissue did not produce characteristic spheres. Node-I tissue formed clusters of cells and did not produce typical sphere contours (Figure 3A, c and d).

### Self-renewal of cancer cell spheres

The abilities for self-renewal and adoption of a spherical morphology, attributed to the presence of CSCs, were tested by collecting the CD44+ cell spheres and culturing them after dissociation into single-cells. The secondary spheres were formed within 1 week from OTSCC samples while small spheres from hyperplastic tissue did not regenerate secondary spheres. Secondary spheres, with typical meroclones and paraclones features, were formed by the third day from non-metastatic and metastatic OTSCC (Figure 3B, a-c), but in notably different numbers 10–15 vs. 15–25, respectively (Table 2). A progressive loss of regenerative ability by the third passage was observed with primary cells from all sources.

## Discussion and conclusion

We established primary cultures from human OTSCC and matched neck nodes Level I, to grow cancer cell spheres, isolated CD44+ cells by MACS followed by culturing to produce CD44+ cell spheres. Moreover, in agreement with the published work [12], spheres in suspension and adherent holoclones, meroclone and paraclone were observed. Epithelial stem cells of endodermal origin have been isolated from mouse tongue previously and their cultures produce self-renewable holoclones [14]. The retention of intrinsic properties restricted to specific sub-population could be used to create a long-lasting response to treatment and prolonged disease free survival by elimination of these by targeted therapy [15].

The cells isolated from hyperplastic or non-cancer tissue did not produce characteristic spheres confirming the hypothesis that only tumor cells have the sub-population of sphere forming cells retain self-renewal abilities. The spheres were created and survived over different time periods (1–21 days), which could be attributed to the different type of cells in various tissues. From the difference in patterns of sphere formation from hyperplastic and carcinoma specimen in cell culture, it is evident that every tumor is individual at cellular level stressing the need for personalized medicine.

CD44 plays various roles from adhesion to signaling [16], and is also known as a marker of tumor aggressiveness playing a functional role in metastasis [17] and a promising marker of CSCs [18]. CD24 is a known marker of differentiation [6] and plays role in B-cell interactions [19]. As observed in this study, the detection of CD44 in the basal-like cell layer, containing tumorigenic population of cancer cells, has been reported in primary head and neck squamous cell cancer specimen [20].

In the present study, the idea was that if some CSCs get trapped in the neck nodes, these will generate the similar spheres in culture as formed in case of tongue tumors. Since neck nodes can be available in large amounts and are more proliferative so would be an ideal *in vitro* system for future studies on CSCs in primary culture. In node, however, only small cell clusters were formed in CD44+ cells cultures (Figure 3A, c) possibly because of low number of these cells or competing markers on various cells for CD44 + ve magnetic beads. We observed that sphere formation is correlated with the nodal involvement of cancer because pathologically node positive OTSCC produced cell spheres in large numbers correlating with worse prognosis. Comparative account of these spheres in various stages of cancer can be studied giving clues regarding progress of disease and metastasis. In head and neck cancer, lymph node metastasis has been linked with poor prognosis and distant metastasis [21]. In order to metastasize, cancer cells must first detach from the primary tumor and invade blood vessels or lymphatics either by a passive process where cells are simply sloughed off from the primary tumor or an active one involving directed migration [22]. While some gene expression studies have suggested that distant metastases resemble their primary tumors of origin [23] other studies have indicated that the expression of specific genes is altered in metastatic cells [24]. It can be assumed that the altered expression of a limited number of genes may render a sub-population of cells fully competent for metastasis, without changing its overall similarity with the primary tumor. A rise in number of circulating CSCs would correlate with the possibility of metastasis. There is an urgent need for information on the possible molecular mechanisms mediating the self-renewal of CSCs by their characterization at the genetic and proteomic level. Comparison of normal and cancer stem cells may provide a starting point for identifying proteins responsible for driving these mechanisms. The results of this preliminary study provide the means for generating CSCs that we will use for in-depth ‘omics analysis.

In summary, we successfully generated cancer cell spheres in primary cultures of OTSCC and neck nodes Level I. The formation of similar cell spheres in node primary cultures as found in OTSCC cultures may offer an opportunity to use this *in vitro* system to characterize CSCs since neck nodes can be available in large amounts and are highly proliferative. We also isolated CD44+ cells from cancer cell spheres by magnetic beads and cultured in supplemented medium producing CD44+ cell spheres. The potential applications for tongue and nodal CSCs characterization include monitoring of CSCs as biomarkers of acquired resistance to new cancer therapies, identifying new potential therapeutic targets to directly suppress cancer metastasis and develop high-throughput technologies for CSC detection applying these at earlier stages of cancer progression with the goal of early cancer detection.
